# Long noncoding RNA PART1 promotes progression of non‐small cell lung cancer cells via JAK‐STAT signaling pathway

**DOI:** 10.1002/cam4.2494

**Published:** 2019-08-22

**Authors:** Dengyan Zhu, Yang Yu, Wei Wang, Kai Wu, Donglei Liu, Yang Yang, Chunyang Zhang, Yu Qi, Song Zhao

**Affiliations:** ^1^ Department of Thoracic Surgery The First Affiliate Hospital of Zhengzhou University Zhengzhou China; ^2^ Department of Anesthesiology The First Affiliated Hospital of Zhengzhou University Zhengzhou China; ^3^ Department of Thoracic Surgery Henan Medical Association Zhengzhou China

**Keywords:** JAK‐STAT, miR‐635, NSCLC, PART1, progression

## Abstract

Non‐small cell lung cancer (NSCLC), the major type of lung cancer, becomes the greatest threat to the life of people. Growing evidence shows prostate androgen‐regulated transcript 1 (PART1) is considered as effective markers for prostate cancer, and has been shown to be associated with poor prognosis of NSCLC. However, the tumorigenic mechanism of PART1 in NSCLC remains to be investigated. In this study, we found that the expression of PART1 was robustly induced in NSCLC tissues and cell lines. Functional studies established that overexpression of PART1 could promote NSCLC cell proliferation, migration, and invasion, while interference of PART1 inhibited NSCLC progression. Our results also identified miR‐635 as a novel target of PART1, whose expression was inhibited by PART1 in NSCLC cell lines. Moreover, gain‐ and loss‐of‐function studies revealed that PART1 could sponge miR‐635 and increase the expression of Janus kinase (JAK) and signal transducer and activator of transcription proteins (STATs). Finally, we deciphered the molecular mechanism by which PART1 contributed to promotion of NSCLC cell progression via phosphorylation and activation of JAK‐STAT signaling pathway. The animal experiment further confirmed that interference of NSCLC could suppress in vivo tumorigenic ability of NSCLC with favorable pharmacological activity via inactivation of JAK‐STAT signaling pathway. In conclusion, our findings clarified the biologic significance of PART1/miR‐635/JAK‐STAT axis in NSCLC progression and provided novel evidence that PART1 may be a new potential therapeutic target for the treatment of NSCLC.

## Graphical abstract

Long noncoding RNA prostate androgen‐regulated transcript 1 (PART1) was induced in non‐small cell lung cancer (NSCLC) tissues and cells. PART1 enhanced proliferation, migration, and invasion of NSCLC cells.

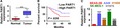

## 1 INTRODUCTION

Lung cancer, caused by a combination of genetic and environmental factors,^1^ is the most common cause of cancer‐related mortality in human.^2^ Due to high metastasis into other tissues,^3^most cases of lung cancer are not curable.^4^ Non‐small cell lung cancer (NSCLC) accounts for about 85% of all lung cancers,^5^ and is usually found at the advanced or metastatic stage.^6^ Despite various therapies, such as surgical resection or chemotherapy for NSCLC,^7^ the 5‐year survival rates for different stages of NSCLC are relatively low and even 1% for stage IV.^8^ Hence, to improve NSCLC treatment, it is important to explore the molecular pathogenesis of NSCLC and discover more effective therapeutic targets for inhibition of the progression.

With the rapid development of molecular biology and gene diagnosis technology, more and more evidences show that long noncoding RNAs (lncRNAs), with 200 nucleotides in length, are closely related to the occurrence of cancer, and thus can be served as a specific tumor biomarker.^9^ Prostate androgen‐regulated transcript 1 (PART1) is a new lncRNA found in prostate tissues and cells via high‐throughput sequencing of RNA,^10^ which is overexpressed in prostate cancer and is beneficial to the proliferation of prostate cancer cells through toll‐like signaling pathway.^11^ Otherwise, PART1 promotes tumor progression of colorectal cancer via sponging miR‐143 and regulating DNA‐methyltransferase 3A.^12^ In esophageal squamous cell carcinoma (ESCC), PART1 is upregulated and involved in poor response to gefitinib treatment, thus functioning as a novel therapeutic target for the disease.^13^ Moreover, previous study has shown that PART1 is upregulated in NSCLC specimens with poor prognosis and is related to tumor recurrence of NSCLC.^14^ However, the regulation mechanism of PART1 in NSCLC has not been studied. Generally, the lncRNAs function in an intricate way,^15^, ^16^ as a competing endogenous RNAs (ceRNAs) to sponging miRNAs to affect target mRNA.^17^ MiR‐635 was first identified in colorectal cancer.^18^ Moreover, it was reported that miR‐635 could inhibit the tumorigenesis of NSCLC by targeting Ying Yang 1 (YY1).^19^ To date, whether PART1 binds with miR‐635 relevant molecular mechanism still needs to be confirmed by further experiments.

As well known, Janus kinase (JAK) and signal transducer and activator of transcription proteins (STATs) signaling pathway play an important role in regulating cell apoptosis, embryonic development, liver regeneration, glycolysis and inflammatory reaction, epithelial mesenchymal transformation as well as angiogenesis.^20^ In various tumor cells, the continuous activation of JAK‐STAT could promote malignant transformation of the tumors.^21^ As for NSCLC, phosphorylation of STAT accounts for 22%‐65% of NSCLC,^22^ which is activated by JAK.^23^ Therefore, JAK‐STAT signaling pathway is a critical mediator of NSCLC.^24^ It was then hypothesized that PART1, miR‐635, and JAK‐STAT might function to modify the progression of NSCLC. In response, the aim of this study was to detect whether lncRNA PART1 regulates progression of NSCLC via targeting JAK‐STAT signaling pathway through sponging of miR‐635.

## 2 MATERIALS AND METHODS

### 2.1 Human NSCLC tissues and animal experimental model

A total of 60 patients diagnosed with NSCLC from The First Affiliate Hospital of Zhengzhou University were recruited in this study. Approval for this study was acquired from Ethics Committee of The First Affiliate Hospital of Zhengzhou University. Written informed consents were acquired from all participants. The NSCLC samples and adjacent normal tissues were acquired from patients immediately after surgical removal. The samples were kept in −80°C freezer for the following experiments. Healthy BALB/c nude mice weighing between 25 and 30 g were used for experimentation, and each mouse was housed separately with standard pellet diet and water. The animal experiments were approved and reviewed by the Institutional Animal Care and Use Committee of The First Affiliate Hospital of Zhengzhou University.

### 2.2 Cell culture

Human lung epithelial cell line (BEAS‐2B), human embryonic kidney 293T (HEK‐293T) cells, and human NSCLC cell lines, A549, H1650, SK‐MES‐1, and H1975, were purchased from American Type Culture Collection (ATCC). All the cells were cultured in DMEM medium containing 10% fetal bovine serum (GIBCO) in incubator at 37°C and under a humidified atmosphere with 5% CO_2_. The cells with confluence of 80% were used for the experiments.

### 2.3 Cell transfection

For the overexpression of PART1, the PART1 primary precursor sequence was polymerase chain reaction (PCR) amplified and cloned into the lentiviral plasmids pLenti‐DEST lentivector (Thermo Fisher). NSCLC cell lines (H1975 and H1650) were seeded at a concentration of 4 × 10^5^ cells per well in 12‐well plates and incubated overnight. After removing the culture medium, the cells were washed with phosphate buffered saline (PBS). The cells were transfected with PART1 lentivirus vector or the empty vector in the presence of ViraPower^™^ Packaging Mix (Thermo Fisher) and 8 mg/mL polybrene.

For the knocking down of PART1, shRNAs were synthesized from Vigenebio, and also cloned in to lentiviral plasmids (PART1‐sh1 and PART1‐sh2). Similarly, NSCLC cell lines (A549 and SK‐MES‐1) were seeded at a concentration of 4 × 10^5^ cells per well in 12‐well plates and incubated overnight. After removing the culture medium, the cells were washed with PBS. The cells were transfected with PART1‐sh1 and PART1‐sh2 or the negative control (scramble) in the presence of ViraPower^™^ Packaging Mix (Thermo Fisher) and 8 mg/mL polybrene.

MiR‐635 mimics, inhibitors, and the negative controls (Control mimics, NC inhibitor) were synthesized by GenePharma. PCR was used to amplify JAK1 and JAK3. Expression plasmids pcDNA3.1 (Invitrogen) were then constructed and sequenced. Cells were transfected with miR‐635 mimics/inhibitor (40 nmol/L) or their NC or pcDNA3.1‐JAK1 or JAK3 via Lipofectamine 2000 (Invitrogen) according to the manufacturer's instructions. Cells were harvested for RNA extraction and further analysis 48 hours after transfection.

### 2.4 Mouse xenograft assay

First, 2 × 10^6^ A549 cells suspended in 150 µL of PBS were subcutaneously injected into the flanks of nude mice to form NSCLC tumor model. The viral supernatants of A549 cells transfected with PART1‐sh were harvested after transduction of lentivirus for 3 days and analyzed the titer at a multiplicity of infection of 5. Then 5 × 10^8^ pfu of viral supernatants were intratumorally injected into the mice twice per week. The tumor volume and weight were measured every week. Approximately 42 days later, the mice were sacrificed with 40 mg/kg sodium pentobarbital. The tumor tissues were isolated, and RNAs and proteins were extracted for analysis.

### 2.5 Histologic evaluation and immunohistochemistry

The NSCLC tumor tissues of different groups of mice were immediately dissected, fixed in 10% buffered formalin, and processed by paraffin tissue processing machine. Seven micrometer sections were stained with hematoxylin and eosin (H&E) for 1 hour. Representative photomicrographs were captured using the microscope (Olympus).

Immunohistochemistry was performed to assess the expression of proteins Ki67 in the tumor tissues. Following deparaffinization and rehydration, sections were blocked for endogenous peroxidase by incubating in 3% H_2_O_2_ and then washed in PBS containing 0.05 mol/L EDTA followed by 4% paraformaldehyde. Tissues were incubated in 4% dry milk and 0.3% goat serum in PBS solution for 20 minutes to block nonspecific binding. Then 4‐μm sections were incubated overnight at room temperature with anti‐Ki67 (Santa Cruz) antibody in the presence of 10% rabbit serum. After washing, sections were then incubated for another 2 hours with horseradish peroxidase (HRP) goat anti‐rabbit IgG secondary antibody. Slides were counterstained with hematoxylin to stain cell nuclei and examined under a light microscope (Olympus).

### 2.6 qRT‐PCR

Sample RNAs were extracted from NSCLC samples and adjacent normal tissues or different cell lines via Trizol reagent (Invitrogen), as well as miRNAs were extracted using miRcute miRNA isolation kit (Tiangen). The RNAs were then reverse transcribed into cDNAs using miScript Reverse Transcription kit (Qiagen). cDNAs were amplified using SYBR1 Premix Ex Taq^™^ II (Takara). U6 was used as the internal reference and GAPDH as the endogenous controls. Three technological replicates were used to ensure the reliability of the analysis. The primer sequences were showed: PART1, 5′‐AAGGCCGTGTCAGAACTCAA‐3′ (forward) and 5′‐GTTTTCCATCTCAGCCTGGA‐3′ (reverse); miR‐635, 5′‐TATAGCATATGCAGGGTG‐3′ (forward) and 5′‐CGCATTCGGAGTGCGAGTT‐3′ (reverse); JAK1, 5′‐GGCTCGTGCGTGTCCTAC‐3′ (forward) and 5′‐GGTCGTCCGCTTATCGTG‐3′ (reverse); JAK3, 5′‐CAGCCCCAACCAGATGTC‐3′ (forward) and 5′‐CCGCTTGATGCCTTTGTAG‐3′ (reverse); U6, 5′‐CTCGCTTCGGCAGCACA‐3′ (forward) and 5′‐AACGCTTCACGAATTTGCGT‐3′ (reverse); GAPDH, 5′‐TGTTCGTCATGGGTGTGAAC‐3′ (forward) and 5′‐ATGGCATGGACTGTGGTCAT‐3′ (reverse).

### 2.7 RNA pull down

1 × 10^7^ H1975 and H1650 cells transfected with PART1 lentivirus vector for the overexpression of PART1 were harvested, 2‐μg cell lysates were mixed with biotinylated PART1 for 2 hours, and then incubated with streptavidin agarose beads at room temperature for 1 hour. The coprecipitated RNAs were purified using TRIZOL and detected by qRT‐PCR.

### 2.8 RNA immunoprecipitation (RIP)

H1975 and H1650 cells transfected with PART1 lentivirus vector for the overexpression of PART1 were collected and lysed, and then incubated with protein G Sepharose beads (GE Healthcare) coated with anti‐AGO2 antibody (Abcam) at 4°C overnight, and anti‐IgG antibody was used as the negative control. RNA was then isolated for qRT‐PCR as mentioned before.

### 2.9 Cell proliferation assay

One hundred microliters 5 × 10^3^ NSCLC cell lines per well were cultured in 96‐well plates for 24 hours and then were treated with Anlotinib for another 24 hours. The 3‐(4, 5‐dimethylthiazol‐2‐yl)‐2, 5‐diphenyl‐tetrazolium bromide (MTT) assay (Solarbio) was utilized to estimate cell viability, and the cell viabilities were calculated by means of spectrophotometric plate reader (BioTek) at 450 nm. All the results were tested at least by three independent experiments.

5‐Ethynyl‐2’‐deoxyuridine (EdU) cell proliferation assay kit (Ribobio) was used to detect the cell proliferation of NSCLC cell lines. Two hundred microliter 2 × 10^4^/mL cultured NSCLC cells were incubated with 50‐µmol/L EdU for 8 hours. After fixation with 70% alcohol for 15 minutes at room temperature and permeabilization with Triton X‐100 for 20 minutes at room temperature, the cells were then incubated with Apollo Staining reaction liquid to labeling the cells. The nuclei were stained with Hoechst 33342. Immunostainings were visualized and photographed with a fluorescent microscope (Olympus inverted microscope IX71).

### 2.10 Flow cytometry

1 × 10^4^ cultured NSCLC cells per well of different treatment were harvested and washed with PBS. The cells were then fixed with 70% ethanol at 4°C for at least 30 minutes. After washing with PBS, ribonuclease (Abcam) was added to the cells, and the propidium iodide (200 µL, Abcam) was used to stain the cells. FACS flow cytometer (Attune, Life Technologies) was used to analyze the cell cycle.

### 2.11 Wound‐healing assay

1 × 10^6^ NSCLC cells were cultured and seeded into 24‐well plates for 24 hours. The wound gaps were then formed via gentle scratching using a plastic pipette tip in the cell monolayer of each well. The monolayers were then washed with PBS to remove debris or the detached cells, and cultured in DMEM for another 24 hours before calculating the wound width under an inverted microscope.

### 2.12 Transwell assay

5 × 10^4^ NSCLC cells per well were seeded into the upper wells of chamber (BD Biosciences) with the Matrigel‐coated membrane (BD Biosciences). Migration‐inducing medium (with 10% FBS) were added to the lower wells of chambers. Eight hours later, the medium of upper wells and the filters were removed. Twenty‐four hours later, the invasive cells to the bottom of chambers were fixed with 100% methanol for 30 minutes, washed with PBS, and then stained with 0.1% crystal violet for 1 hour. The stained cells were imaged and counted under microscope (Olympus).

### 2.13 Dual‐luciferase reporter assay

The fragments of wild‐type 3′UTR of JAK1, JAK3 (JAK1‐wt, JAK3‐wt) containing the binding sites for miR‐635 were amplified and cloned into psi‐CHECK^™^‐2 vector (Promega), respectively, as well as the mutant JAK1, JAK3 (JAK1‐mut, JAK3‐mut) whose binding ability with miR‐635 were lost. 3 × 10^4^ HEK‐293T cells per well were seeded in 48‐well plates for 24 hours. Cells were then transfected with psiCHECK^™^‐2‐JAK1‐wt/JAK3‐wtor psiCHECK^™^‐2‐JAK1‐mut/JAK3‐mut in combination with miR‐635 mimics (80 nmol/L; GenePharma) via Lipofectamine 2000 (Invitrogen). Forty‐eight hours later, luciferase activities were measured using the Dual‐Luciferase Reporter Assay System (Promega) and detected by lumat LB 9501 luminator (EG&G Berthold). Firefly luciferase activity was normalized to Renilla luciferase activity for each group.

### 2.14 Western blot

NSCLC tissues, cultured, or transfected cells were harvested and lysed in RIPA buffer (KeyGen). Protein lysates were loaded onto 10% SDS‐PAGE, and then transferred to PVDF membrane. The membrane was blocked in PBS‐T with 5% bovine serum albumin for 1 hour. PVDF membranes were then probed with rabbit anti‐p21, p27, JAK1, JAK3, STAT3 monoclonal antibody (1:1000, Abcam), Cyclin D2, E‐cadherin, p‐JAK1, p‐JAK3, p‐STAT3 (1:1500, Abcam), MMP2, MMP9, tissue inhibitor of metalloproteinases (TIMP‐1), proto‐oncogene serine/threonine‐protein kinase (Pim‐1), GAPDH (1:2000, Abcam) overnight at 4°C. The PVDF membrane was washed with TBST and labeled with HRP‐conjugated secondary antibodies (1:3000; Abcam) for 1 hour. Immunoreactivities were detected by enhanced chemiluminescence (KeyGen). GAPDH was used as a control.

### 2.15 Statistical analysis

All results are expressed as mean ± SEM of at least three independent experiments. By means of GraphPad Prism software (GraphPad Prism Software Inc) and one‐way analysis of variance, we determined the statistical analyses. *P* < .05, *P* < .01, or *P* < .001 was considered as a mark of statistically significant.

## 3 RESULTS

### 3.1 LncRNA PART1 was induced in NSCLC tissues and cells

**Figure 1:**
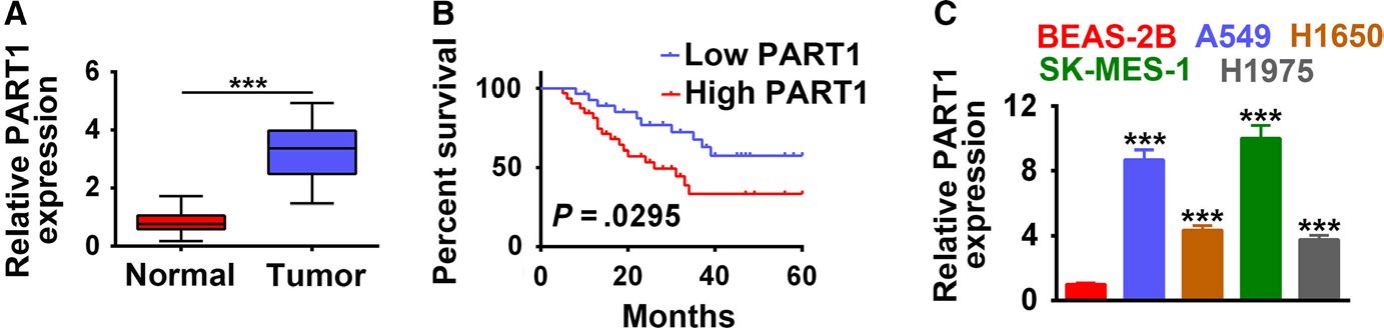
LncRNA PART1 was induced in NSCLC tissues and cells. A, Expression of PART1 in 60 paired NSCLC specimens and adjacent normal tissues. ***P < .001. B, Overall survival of NSCLC patients subdivided by PART1 expression levels. C, Expression of PART1 in NSCLC cell lines A549, H1650, SK‐MES‐1, and H1975 and control cell line, BEAS‐2B. ***P < .001. LncRNA, long noncoding RNA; NSCLC, non‐small cell lung cancer; PART1, prostate androgen‐regulated transcript 1

As shown in Figure 1A, PART1 was markedly upregulated in human NSCLC tissues compared to adjacent normal tissues (n = 60, *P* < .001), suggesting potential relation between PART1 and pathogenesis of NSCLC. Further refinement analysis of correlation between PART1 expression and clinic pathologic characteristics of NSCLC patients showed that among the 60 patients, high expression of PART1 (fold change ≥2.5) comprised 32 persons (Table 1). Moreover, high expression of PART1 was more frequently observed in squamous NSCLC tumors (*P* = .031) (Table 1). The age (*P* = .5), gender (*P* = .605), smoking status (*P* = .448), tumor differentiation (*P* = .916), or epidermal growth factor receptor (EGFR) mutations (*P* = .886) showed no significant correlation with PART1 expression (Table 1). Kaplan‐Meier analysis revealed that patients with high expression of PART1 had lower overall survival (OS) rate than those with low expression of PART1 (*P* = .0295, Figure 1B). Moreover, as shown in Table 2, univariate analysis showed that histology (*P* = .05) and EGFR mutation (*P* = .04) were significantly related to poor OS of NSCLC. Multivariate analysis showed that independent of histology, PART1 expression levels were a significant prognostic factor for poorer OS of NSCLC. We also identified the expression profiles of PART1 in various NSCLC cell lines.The results indicated that PART1 was significantly upregulated in NSCLC cell lines, A549, H1650, SK‐MES‐1, and H1975, in comparison with the control cells (human lung epithelial cell line, BEAS‐2B; *P* < .001, Figure 1C). In general, the expression files of PART1 in NSCLC tissues and cell lines suggested that PART1 may be involved in the tumorigenesis and development of NSCLC.

**Table 1:** Relation between NSCLC patients’ characteristics and PART1 expression

Factors	Tissue PART1 (n = 60)		*P*
	Low (n = 28)	High (n = 32)	
Age (y)			
≤65	12	11	.5
>65	16	21	
Gender			
Male	13	17	.605
Female	15	15	
Smoking status			
Nonsmoker	13	18	.448
Ever‐smoker	15	14	
Histologic type			
Squamous	8	18	.031
Nonsquamous	20	14	
T status			
T1‐2	16	15	.427
T3‐4	12	17	
N status			
N0	16	18	.944
N1‐3	12	14	
Stage			
Ⅰ‐Ⅱ	15	17	.972
Ⅲ	13	15	
Differentiation			
Well moderate	17	19	.916
Poor	11	13	
EGFR mutation status			
Mutated	10	12	.886
Wild‐type	18	20	

Abbreviations: EGFR, epidermal growth factor receptor; NSCLC, non‐small cell lung cancer; PART1, prostate androgen‐regulated transcript 1.

**Table 2:** Univariate and multivariate analyses for prognostic factors in NSCLC patients

Variables	Univariate			Multivariate		
	HR	95% Cl	*P*	HR	95% Cl	*P*
Age, y (>65 vs ≤65)	0.97	0.45‐2.09	.93			
Gender (male vs female)	0.54	0.26‐1.15	.54			
Smoking (no vs yes)	0.59	0.28‐1.27	.18			
Histology (squamous vs nonsquamous)	2.22	1‐4.93	.05	2.49	1.12‐5.56	.026
T status (T3‐4 vs T1‐2)	1.34	0.64‐2.82	.44			
N status (N1‐3 vs N0)	1.65	0.78‐3.48	.19			
Stage Ⅲ vs Ⅰ‐Ⅱ	0.57	0.26‐1.27	.17			
Differentiation (poor vs well moderate)	1.51	0.68‐3.35	.31			
EGFR mutation status (mutated vs wild‐type)	2.27	1.04‐4.95	.04	2.53	1.15‐5.57	.02

Abbreviations: CI, confidence interval; EGFR, epidermal growth factor receptor; HR, hazard ratio; NSCLC, non‐small cell lung cancer.

### 3.2 PART1 enhanced proliferation, migration, and invasion of NSCLC cells

**Figure 2:**
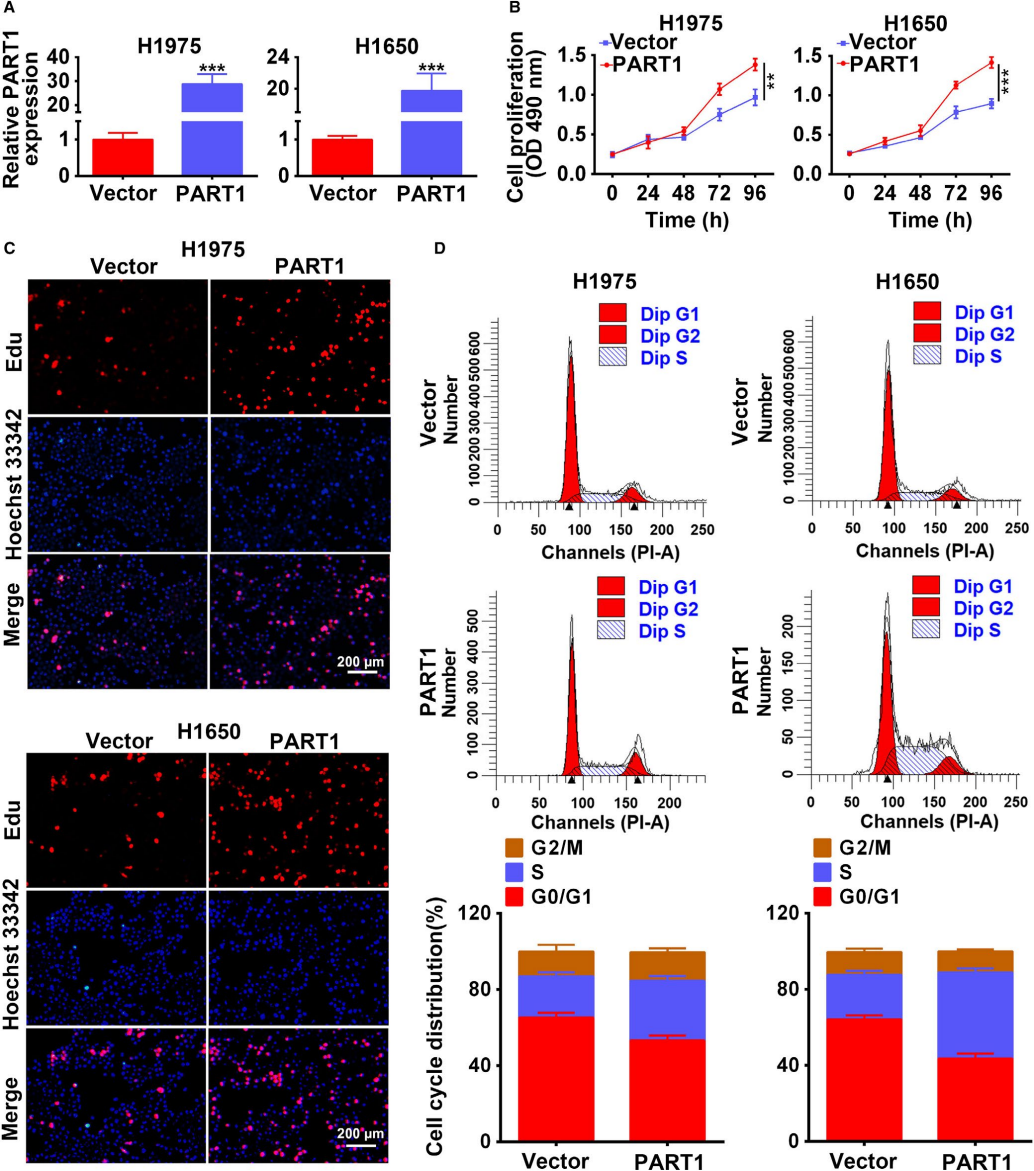
PART1 enhanced proliferation and cell cycle of NSCLC cells. A, Transfection efficiency of PART1 overexpression (PART1) in H1975 and H1650 cells. ***P < .001. B, Detection of PART1 on the cell viability by MTT assay. **, ***P < .01, *P* < .001. C, Detection of PART1 on the cell proliferation by EdU staining. Scale bar: 200 μm. D, Detection of PART1 on the cell cycle by flow cytometry. EdU, 5‐Ethynyl‐2′‐deoxyuridine; MTT, 3‐(4,5‐dimethylthiazol‐2‐yl)‐2,5‐diphenyl‐tetrazolium bromide; NSCLC, non‐small cell lung cancer; PART1, prostate androgen‐regulated transcript 1

**Figure 3:**
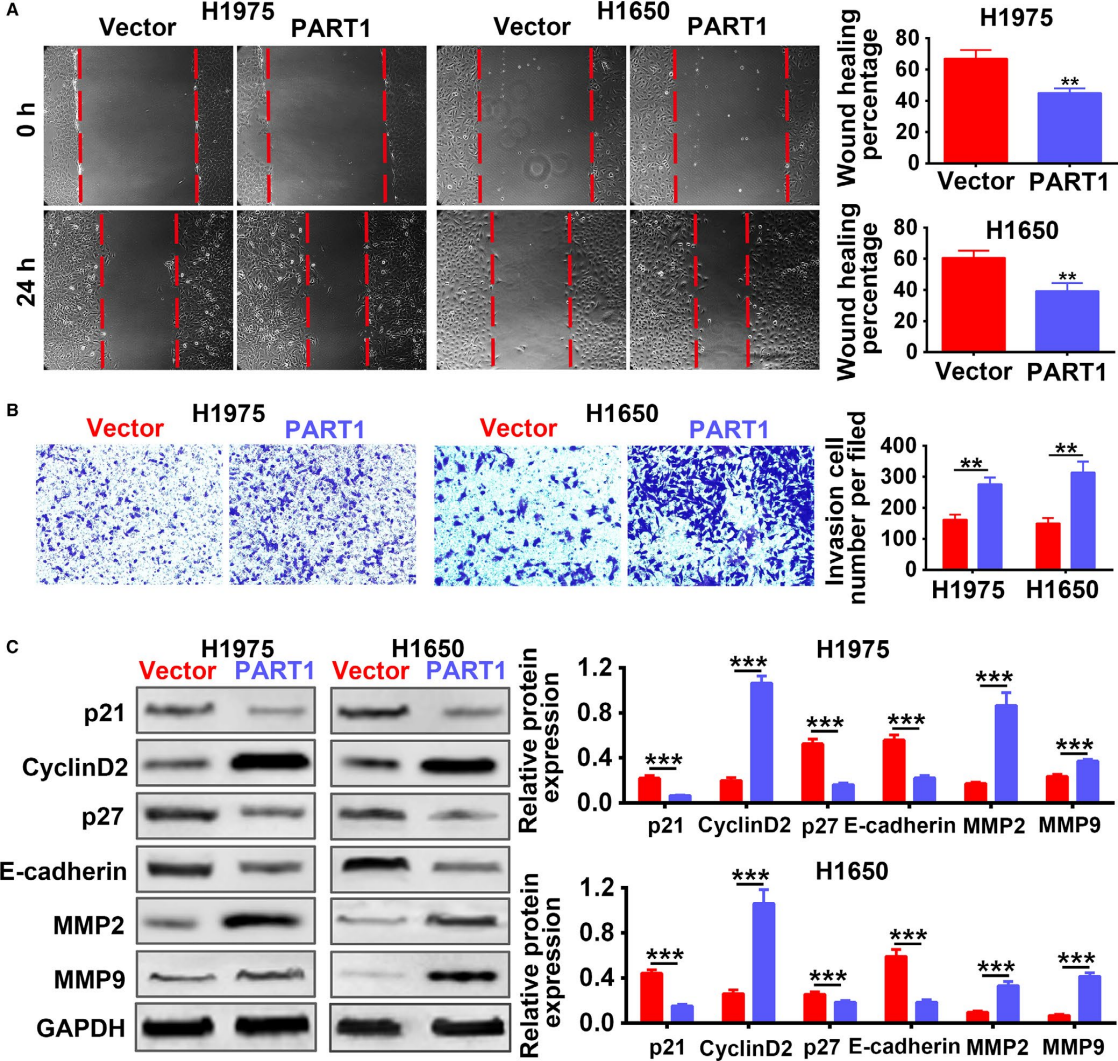
PART1 enhanced migration and invasion of NSCLC cells. A, Detection of PART1 on the migration by wound‐healing assay. **P < .01. B, Detection of PART1 on the invasion by transwell assay. **P < .01. C, Detection of PART1 on the protein expression of p21, Cyclin D2, p27, E‐cadherin, MMP2, and MMP9 by western blot. ***P < .001. NSCLC, non‐small cell lung cancer; PART1, prostate androgen‐regulated transcript 1

Stable H1975 and H1650 cell lines with overexpression of PART1 were established to explore the effect of PART1 on the progression of NSCLC. The transfection efficiency was confirmed in Figure 2A. MTT assay (Figure 2B) and EdU staining (Figure 2C) showed that overexpression of PART1 promoted cell proliferation. Moreover, PART1 induced cell cycle progression from G1 to S phase (Figure 2D). These results revealed that PART1 positively regulated the cell viability and proliferation of NSCLC cells. Moreover, overexpression of PART1 not only promoted cell migration (Figure 3A), but also promoted cell invasion (Figure 3B). Proteins involved in the cell cycle, migration, and invasion were also detected in the study. As shown in Figure 3C, PART1 decreased the expression of p21 and p27, while increased that of Cyclin D2, promoting the cell cycle. Moreover, PART1 decreased the expression of E‐cadherin, increased MMP2 and MMP9, facilitating for the migration and invasion of NSCLC (Figure 3C). All these data indicated that PART1 enhanced proliferation, migration, and invasion of NSCLC cells.

### 3.3 Knockdown of PART‐1 suppressed proliferation, migration, and invasion of NSCLC cells

**Figure 4:**
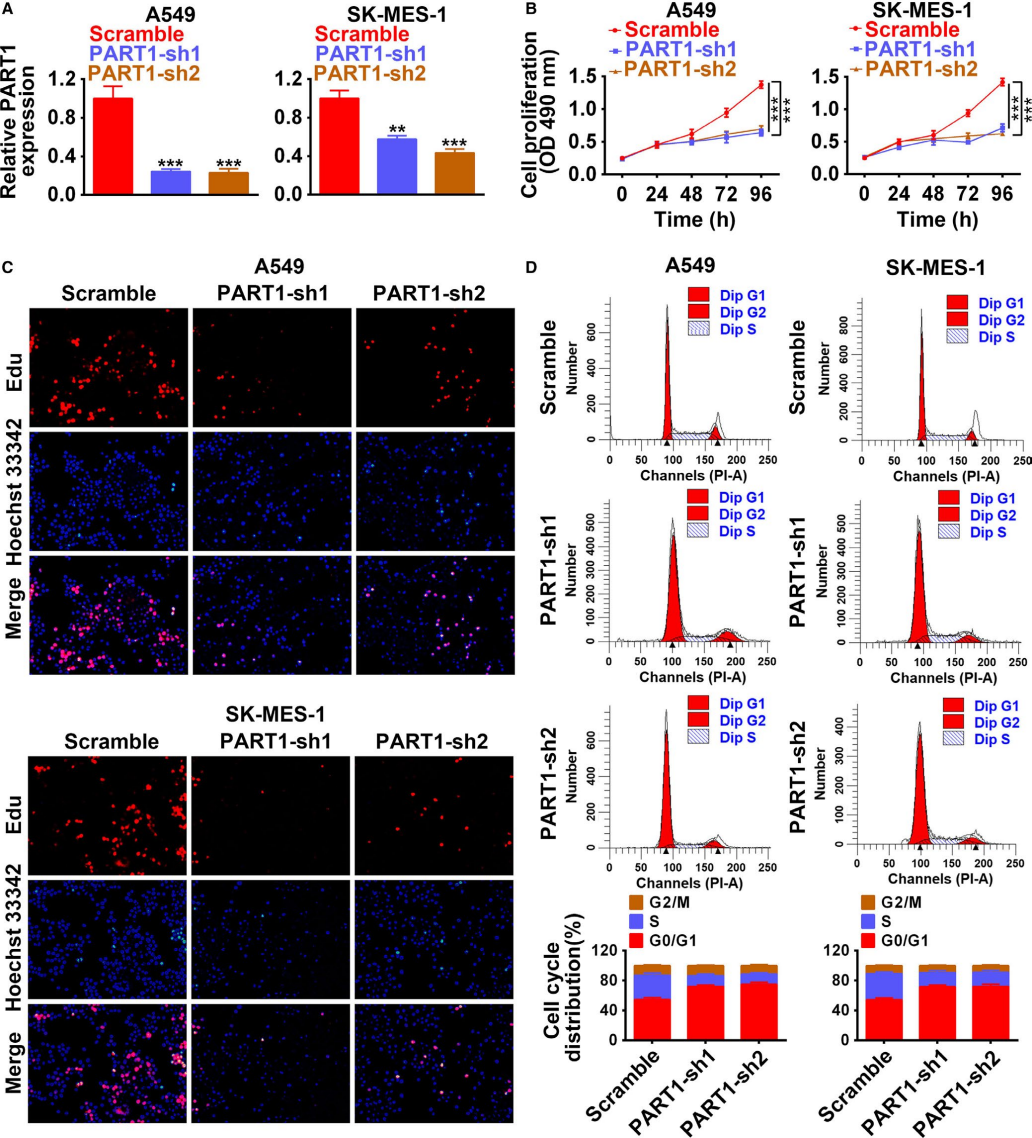
Knockdown of PART‐1 suppressed proliferation and cell cycle of NSCLC cells. A, Transfection efficiency of PART1 shRNAs (PART1‐sh1/sh2) in A549 and SK‐MES‐1 cells. **, ***P < .01, *P* < .001. B, Detection of PART1‐sh1/sh2 on the cell viability by MTT assay. ***P < .001. C, Detection of PART1‐sh1/sh2 on the cell proliferation by EdU staining. D, Detection of PART1‐sh1/sh2 on the cell cycle by flow cytometry. EdU, 5‐Ethynyl‐2’‐deoxyuridine; MTT, 3‐(4,5‐dimethylthiazol‐2‐yl)‐2,5‐diphenyl‐tetrazolium bromide; NSCLC, non‐small cell lung cancer; PART1, prostate androgen‐regulated transcript 1

**Figure 5:**
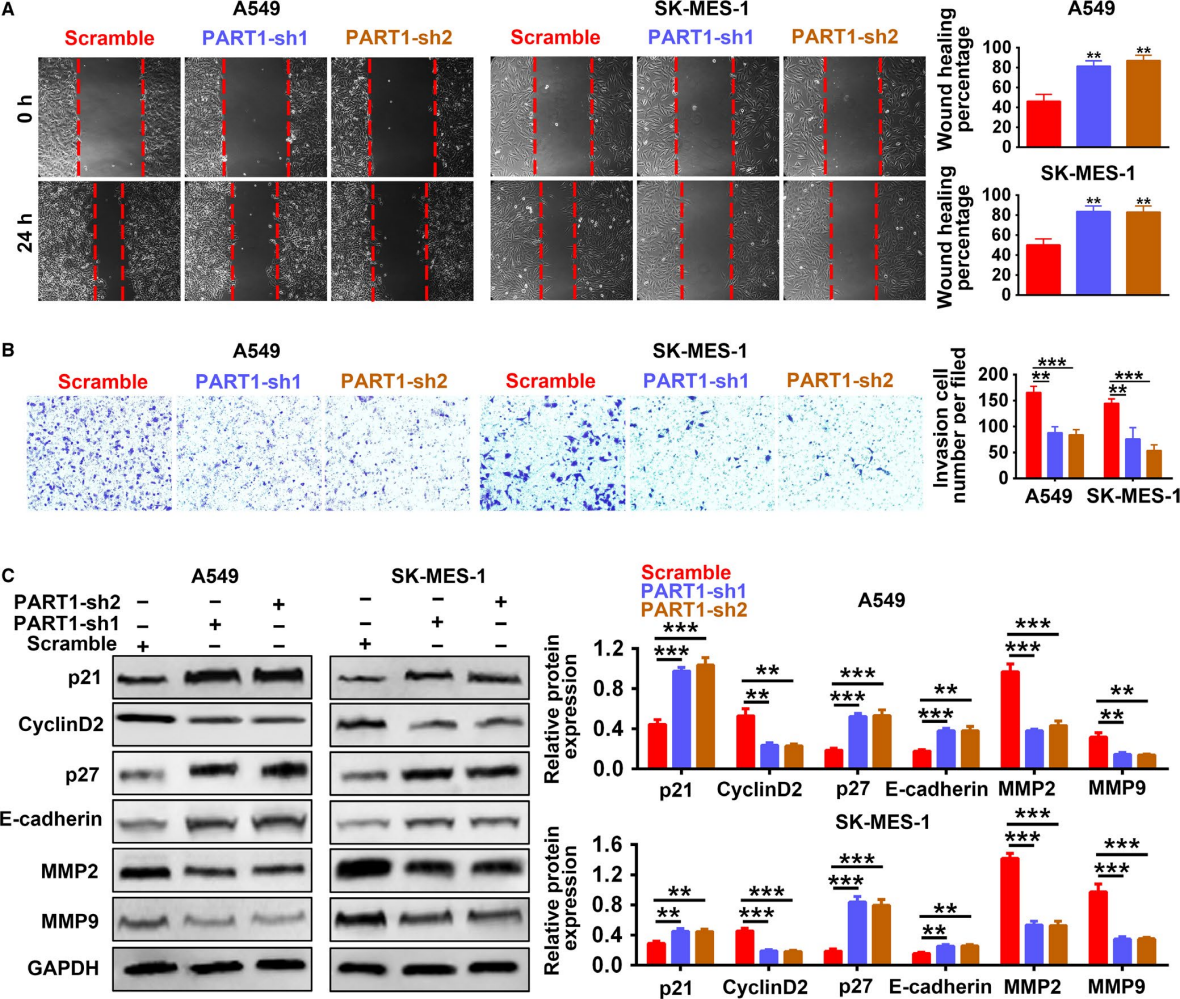
Knockdown of PART‐1 suppressed migration and invasion of NSCLC cells. A, Detection of PART1‐sh1/sh2 on the migration by wound‐healing assay. **P < .01. B. Detection of PART1‐sh1/sh2 on the invasion by transwell assay. **, ***P < .01, *P* < .001. C, Detection of PART1‐sh1/sh2 on the protein expression of p21, Cyclin D2, p27, E‐cadherin, MMP2, and MMP9 by western blot. **, ***P < .01, *P* < .001. NSCLC, non‐small cell lung cancer; PART1, prostate androgen‐regulated transcript 1

Stable A549 and SK‐MES‐1 cell lines with knockdown of PART1 via shRNA (PART1‐sh1, PART1‐sh2) were established. The transfection efficiency was confirmed in Figure 4A with downregulation of PART1 by both PART1‐sh1 and PART1‐sh2. MTT assay (Figure 4B) and EdU staining (Figure 4C) showed that knockdown of PART1 inhibited cell proliferation. Moreover, PART1‐sh1 or sh2 suppressed the cell cycle progression, and arrested the cell at G0/G1 phase (Figure 4D). These results revealed that knockdown of PART1 negatively regulated the cell viability and proliferation of NSCLC cells. Migration and invasion results indicated that knockdown of PART1 not only inhibited cell migration (Figure 5A), but also suppressed cell invasion (Figure 5B). In contrast to overexpression of PART1, knockdown of PART1 increased the expression of p21 and p27, while decreased that of Cyclin D2, thus inhibiting the cell cycle. On the other hand, knockdown of PART1 increased the expression of E‐cadherin, while decreased MMP2 and MMP9, failing for the migration and invasion of NSCLC (Figure 5C). All these data indicated that knockdown of PART‐1 suppressed proliferation, migration, and invasion of NSCLC cells.

### 3.4 PART1 directly bound to and inhibited miR‐635 expression

**Figure 6:**
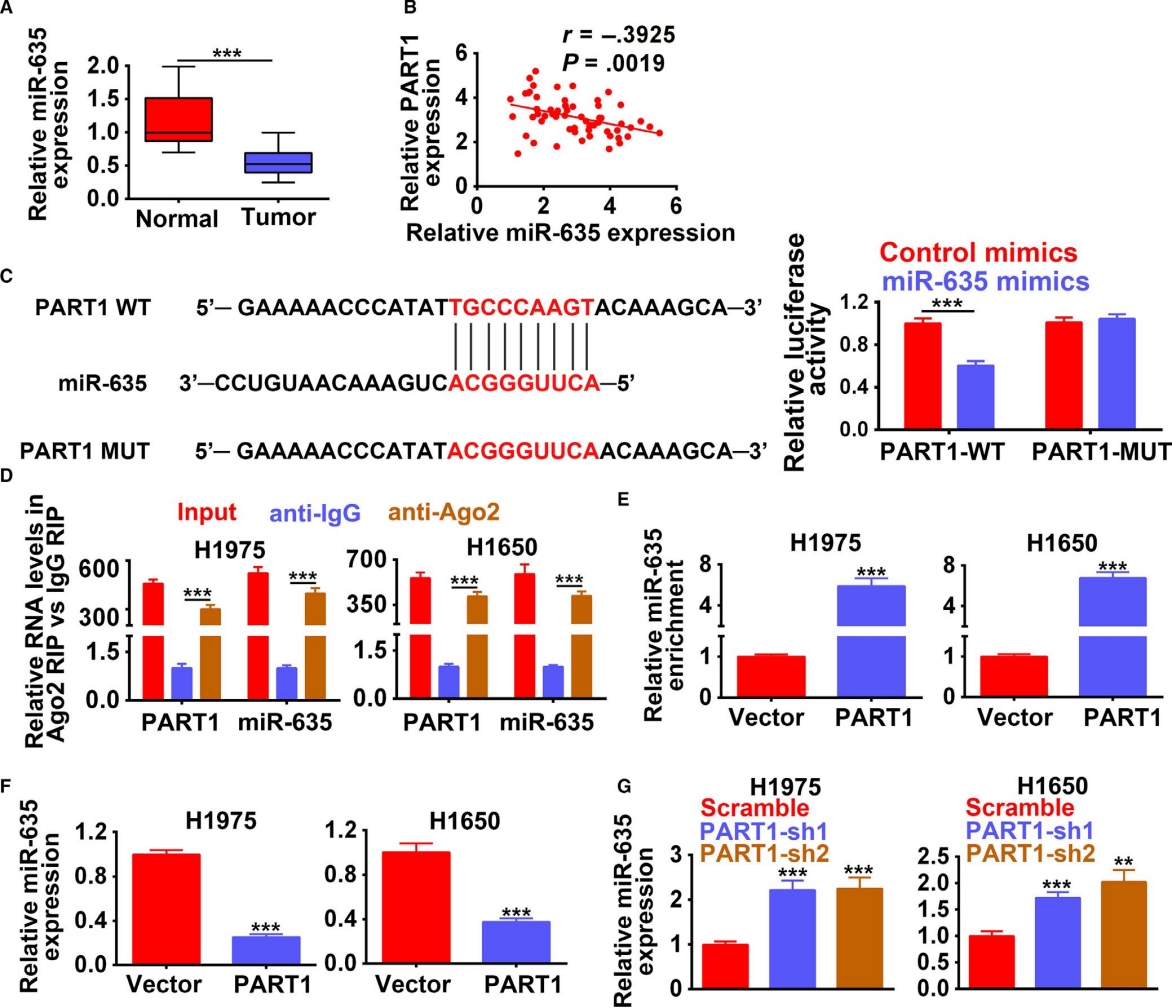
PART1 directly bound to and inhibited miR‐635 expression. A, Expression of miR‐635 in 60 paired NSCLC specimens and adjacent normal tissues. ***P < .001. B, Negative relation analysis between PART1 and miR‐635 expression in NSCLC patients. C, Potential binding site of miR‐635 in PART1. Detection of miR‐635 mimics on luciferase activity of wild‐type or mutant PART1 by luciferase reporter assay. ***P < .001. D, Detection of PART1 on the enrichment of miR‐635 by RIP assay. ***P < .001. E, Detection of PART1 on the enrichment of miR‐635 by RNA pull down assay. ***P < .001. F, Detection of PART1 on the expression of miR‐635 by qRT‐PCR. ***P < .001. G, Detection of PART1‐sh1/sh2 on the expression of miR‐635 by qRT‐PCR. **, ***P < .01, *P* < .001. NSCLC, non‐small cell lung cancer; PART1, prostate androgen‐regulated transcript 1; PCR, polymerase chain reaction; RIP, RNA immunoprecipitation

As shown in Figure 6A, the relative expression of miR‐635 was markedly decreased in human NSCLC tissues compared to adjacent normal tissues (n = 60, *P* < .001), suggesting negative relation between PART1 and miR‐635 in NSCLC (Figure 6B). Further refinement analysis showed that high expression of miR‐635 comprised 22 persons (Table 3). Moreover, high expression of miR‐635 was more frequently observed in nonsquamous NSCLC tumors (*P* = .037) (Table 3), just the contrary to that of PART1. The age (*P* = .651), gender (*P* = .554), smoking status (*P* = .886), tumor differentiation (*P* = .928), or EGFR mutations (*P* = .284) showed no significant correlation with miR‐635 expression (Table 3).

**Table 3:** Relation between NSCLC patients’ characteristics and miR‐635 expression

Factors	Tissue miR‐635 (n = 60)		*P*
	Low (n = 38)	High( n = 22)	
Age (y)			
≤65	15	10	.651
>65	23	12	
Gender			
Male	16	11	.554
Female	22	11	
Smoking status			
Nonsmoker	20	12	.886
Ever‐smoker	18	10	
Histologic type			
Squamous	26	9	.037
Nonsquamous	12	13	
T status			
T1‐2	19	12	.734
T3‐4	19	10	
N status			
N0	17	9	.773
N1‐3	21	13	
Stage			
Ⅰ‐Ⅱ	18	12	.592
Ⅲ	20	10	
Differentiation			
Well moderate	16	9	.928
Poor	22	13	
EGFR mutation status			
Mutated	17	13	.284
Wild‐type	21	9	

Abbreviations: EGFR, epidermal growth factor receptor; NSCLC, non‐small cell lung cancer.

According to starbase analysis, we proposed that PART1 may harbor potential binding site for miR‐635 (Figure 6C). Moreover, luciferase reporter assay showed that miR‐635 mimics dramatically decreased luciferase activity of reporter gene with wild‐type PART1 compared with that of negative control (*P* < .001) (Figure 6C), while the regulatory effect of miR‐635 mimics was suppressed when the predicted miR‐635‐binding site in PART1 was mutated (Figure 6C). RIP demonstrated a strong enrichment of miR‐635 in both H1975 and H1650 (Figure 6D) transfected with overexpression of PART1. RNA pull down also revealed enrichment of miR‐635 via overexpression of PART1 (Figure 6E), confirming the binding ability between PART1 and miR‐635. Moreover, miR‐635 was downregulated by overexpression of PART1 (Figure 6F), upregulated by knockdown of PART1 (Figure 6G). In general, PART1 directly bound to and inhibited miR‐635 expression in NSCLC cells.

### 3.5 JAK1 and JAK3 were direct target genes of miR‐635

**Figure 7:**
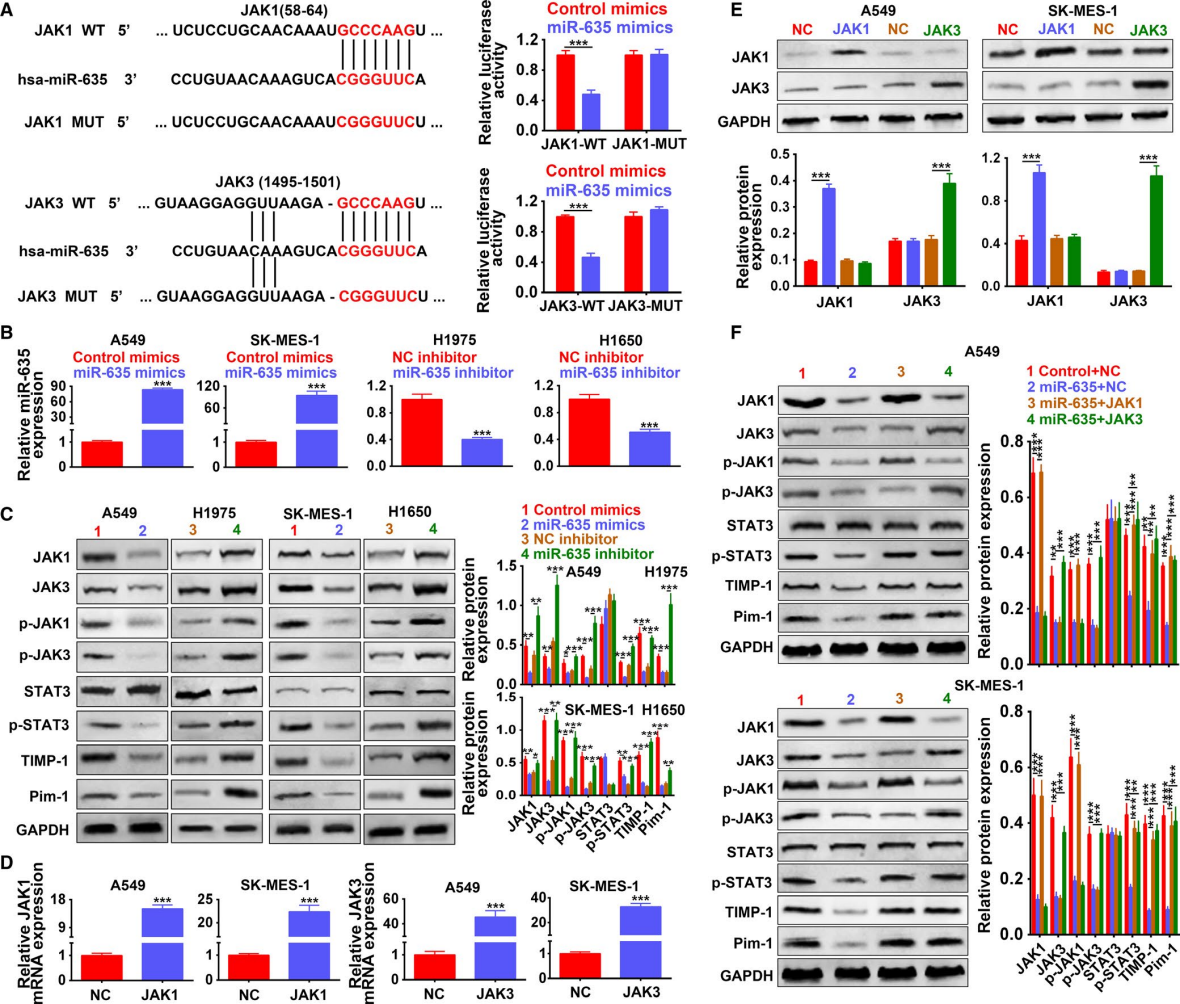
JAK1 and JAK3 were direct target genes of miR‐635. A, Potential binding site of miR‐635 in 3′UTR of JAK1 or JAK3. The mutants were also shown. Detection of miR‐635 mimics on activity of wild‐type or mutant 3′UTR of JAK1 and JAK3 by dual‐luciferase reporter assay. ***P < .001. B, Detection of miR‐635 mimics or inhibitors on expression of miR‐635 by qRT‐PCR, respectively. ***P < .001. C, Detection of miR‐635 mimics or inhibitors on expression of JAK1, JAK3, p‐JAK1, p‐JAK3, STAT3, p‐STAT3, TIMP‐1, and Pim‐1 by western blot. *, **, ***P < .05, *P* < .01, *P* < .001. D, Detection of JAK1 or JAK3 overexpression (JAK1 or JAK3) on the mRNA expression of JAK1 or JAK3 by qRT‐PCR. ***P < .001. E, Detection of JAK1 or JAK3 on the protein expression of JAK1 or JAK3 by western blot. ***P < .001. F, Detection of miR‐635 mimics and JAK1 or JAK3 on expression of JAK1, JAK3, p‐JAK1, p‐JAK3, STAT3, p‐STAT3, TIMP‐1, and Pim‐1. **, ***P < .01, *P* < .001. JAK, Janus kinase; PCR, polymerase chain reaction; Pim‐1, proto‐oncogene serine/threonine‐protein kinase; STAT, signal transducer and activator of transcription protein; TIMP‐1, tissue inhibitor of metalloproteinases

The downstream target mRNA for miR‐635 was predicted as 3′UTR of JAK1 and 3 via Targetscan (Figure 7A). Luciferase reporter assay revealed that miR‐635 mimics significantly inhibited luciferase activity of reporter gene with wild‐type JAK1 or JAK3 3′UTR compared with control mimics (*P* < .001) (Figure 7A), while the luciferase activities of vectors containing the mutant JAK1 or JAK3 3′UTR were not affected by miR‐635 mimics (Figure 7A).These data suggest that miR‐635 directly binds to JAK1 or JAK3 3′UTR. Moreover, results confirmed the transfection efficiency of miR‐635 mimics in A549 and SK‐MES‐1 cells with upregulation (Figure 7B), miR‐635 inhibitor in H1975 or H1650 cells with downregulation (Figure 7B). The protein expression of JAK1 and JAK3 was demonstrated as decreased by miR‐635 mimics and increased by miR‐635 inhibitor (Figure 7C). Results also showed that miR‐635 mimics decreased the phosphorylation of JAK1, JAK3, and STAT3, while miR‐635 inhibitor increased that (Figure 7C), suggesting the inactivation of JAK‐STAT via miR‐635. Downstream genes of JAK‐STAT signaling pathway, TIMP‐1, and Pim‐1, involved in cell cycle progression, were inhibited by miR‐635 mimics and induced by miR‐635 inhibitor (Figure 7C), suggesting the regulation ability of miR‐635 on NSCLC progression. Transfection efficiency of pcDNA‐JAK1/JAK3 for the overexpression of JAK1 and JAK3 was confirmed in both A549 and SK‐MES‐1 cells (Figure 7D) and western blot (Figure 7E). Results showed that addition of pcDNA‐JAK1 or pcDNA‐JAK3 increased the expression of p‐STAT3, TIMP‐1 and Pim‐1 decreased by miR‐635, activating JAK‐STAT signaling pathway (Figure 7F). In conclusion, JAK1 and JAK3 were direct target genes of miR‐635, and miR‐635 was involved in the regulation of JAK‐STAT3 signaling pathway.

### 3.6 PART1 functioned as ceRNA of miR‐635 to activate JAK‐STAT signaling pathway

**Figure 8:**
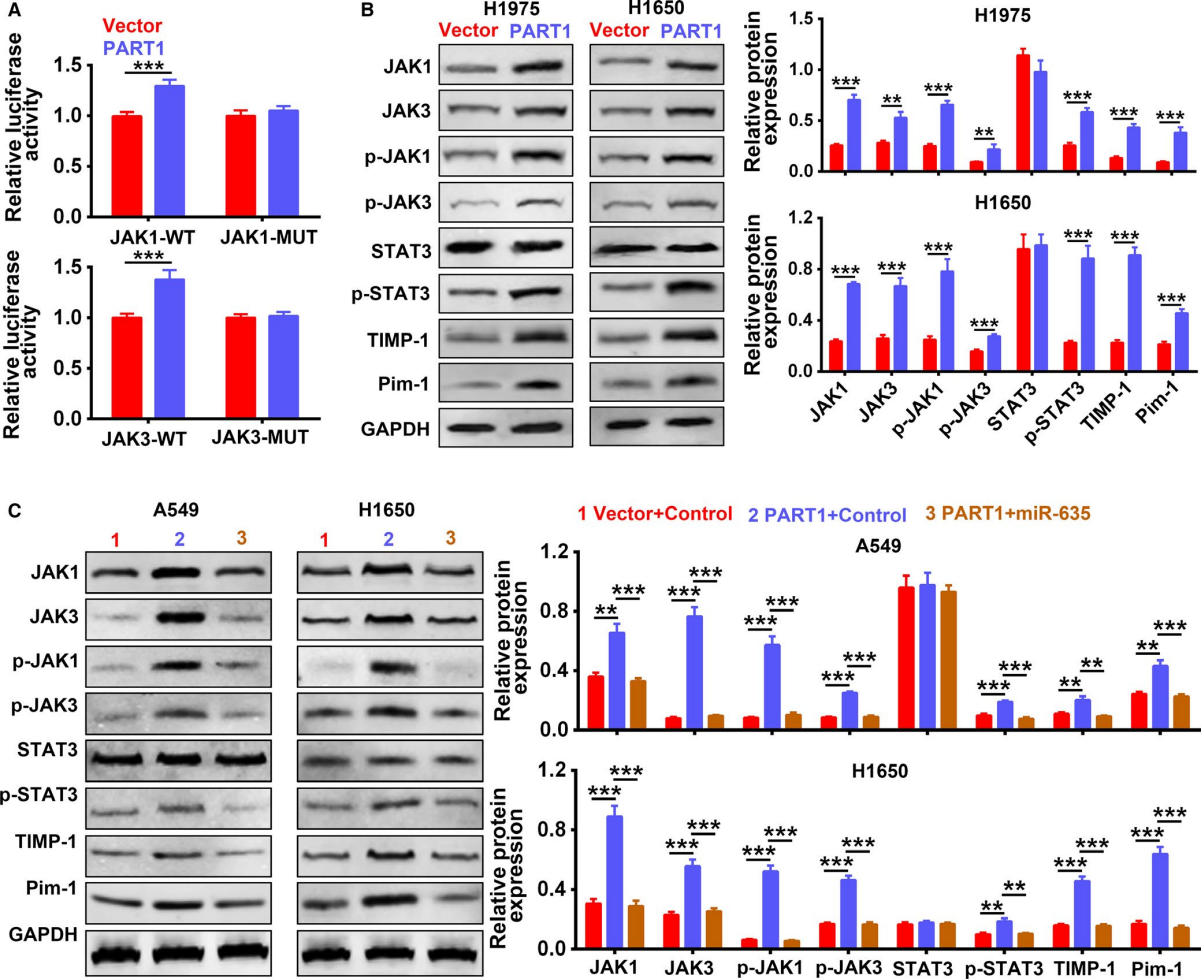
PART1 functioned as ceRNA of miR‐635 to activate JAK‐STAT signaling pathway. A, Detection of PART1 on activity of wild‐type or mutant 3′UTR of JAK1 and JAK3 by dual‐luciferase reporter assay. ***P < .001. B, Detection of PART1 on expression of JAK1, JAK3, p‐JAK1, p‐JAK3, STAT3, p‐STAT3, TIMP‐1, and Pim‐1 by western blot. **, ***P < .01, *P* < .001. C, Detection of PART1 and miR‐635 mimics on expression of JAK1, JAK3, p‐JAK1, p‐JAK3, STAT3, p‐STAT3, TIMP‐1, and Pim‐1 by western blot. **, ***P < .01, *P* < .001. ceRNA, competing endogenous RNA; JAK, Janus kinase; PART1, prostate androgen‐regulated transcript 1; Pim‐1, proto‐oncogene serine/threonine‐protein kinase; STAT, signal transducer and activator of transcription protein; TIMP‐1, tissue inhibitor of metalloproteinases

First of all, overexpression of PART1 increased the luciferase activity of reporter gene with wild‐type JAK1 and JAK3 3′UTR, while had no significant effect on the mutant JAK1 and JAK3 3′UTR (Figure 8A), revealing the vital regulation of PART1 on JAK‐STAT signaling pathway. H1975 and H1650 with overexpressed PART1 increased the expression of JAK1, JAK3, p‐JAK1, p‐JAK3, p‐STAT3, TIMP‐1, and Pim‐1, further confirming the regulation of PART1 on JAK‐STAT signaling pathway (Figure 8B). Moreover, results showed that the upregulation of JAK1, JAK3, p‐JAK1, p‐JAK3, p‐STAT3, TIMP‐1, and Pim‐1 via overexpression of PART1 was inhibited by the addition of miR‐635 mimics (Figure 8C), suggesting that PART1 might function as ceRNA of miR‐635 to activate JAK‐STAT signaling pathway.

### 3.7 PART1 enhanced proliferation, migration, and invasion of NSCLC cells via JAK‐STAT signaling pathway

**Figure 9:**
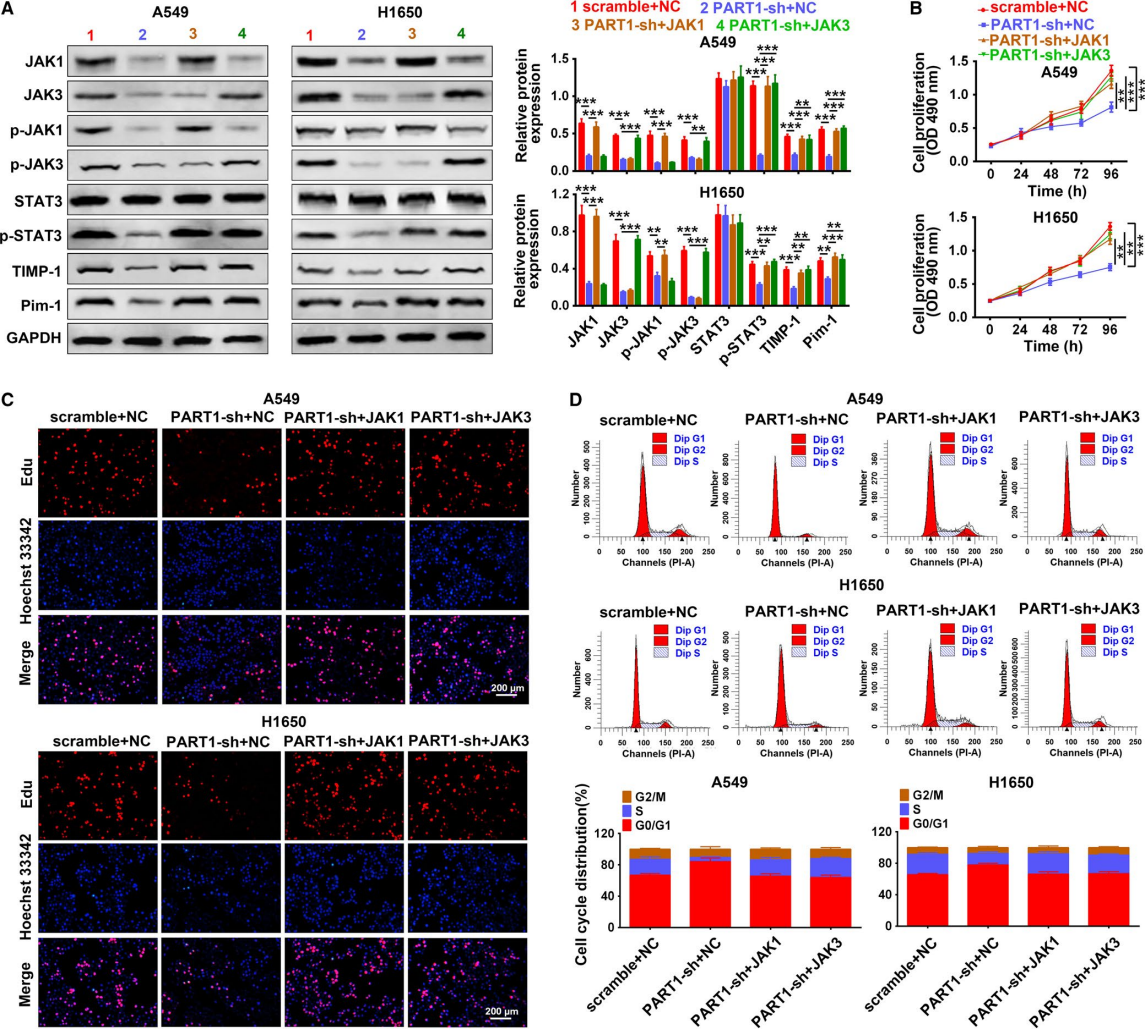
PART1 enhanced proliferation and cell cycle of NSCLC cells via JAK‐STAT signaling pathway. A, Detection of PART1‐sh and JAK1 or JAK3 on expression of JAK1, JAK3, p‐JAK1, p‐JAK3, STAT3, p‐STAT3, TIMP‐1, and Pim‐1 by western blot. **, ***P < .01, *P* < .001. B, Detection of PART1‐sh and JAK1 or JAK3 on cell viability by MTT assay. **, ***P < .01, *P* < .001. C, Detection of PART1‐sh and JAK1 or JAK3 on cell proliferation by EdU staining. D, Detection of PART1‐sh and JAK1 or JAK3 on cell cycle by flow cytometry. EdU, 5‐Ethynyl‐2′‐deoxyuridine; JAK, Janus kinase; MTT, 3‐(4,5‐dimethylthiazol‐2‐yl)‐2,5‐diphenyl‐tetrazolium bromide; NSCLC, non‐small cell lung cancer; PART1, prostate androgen‐regulated transcript 1; Pim‐1, proto‐oncogene serine/threonine‐protein kinase; STAT, signal transducer and activator of transcription protein; TIMP‐1, tissue inhibitor of metalloproteinases

**Figure 10:**
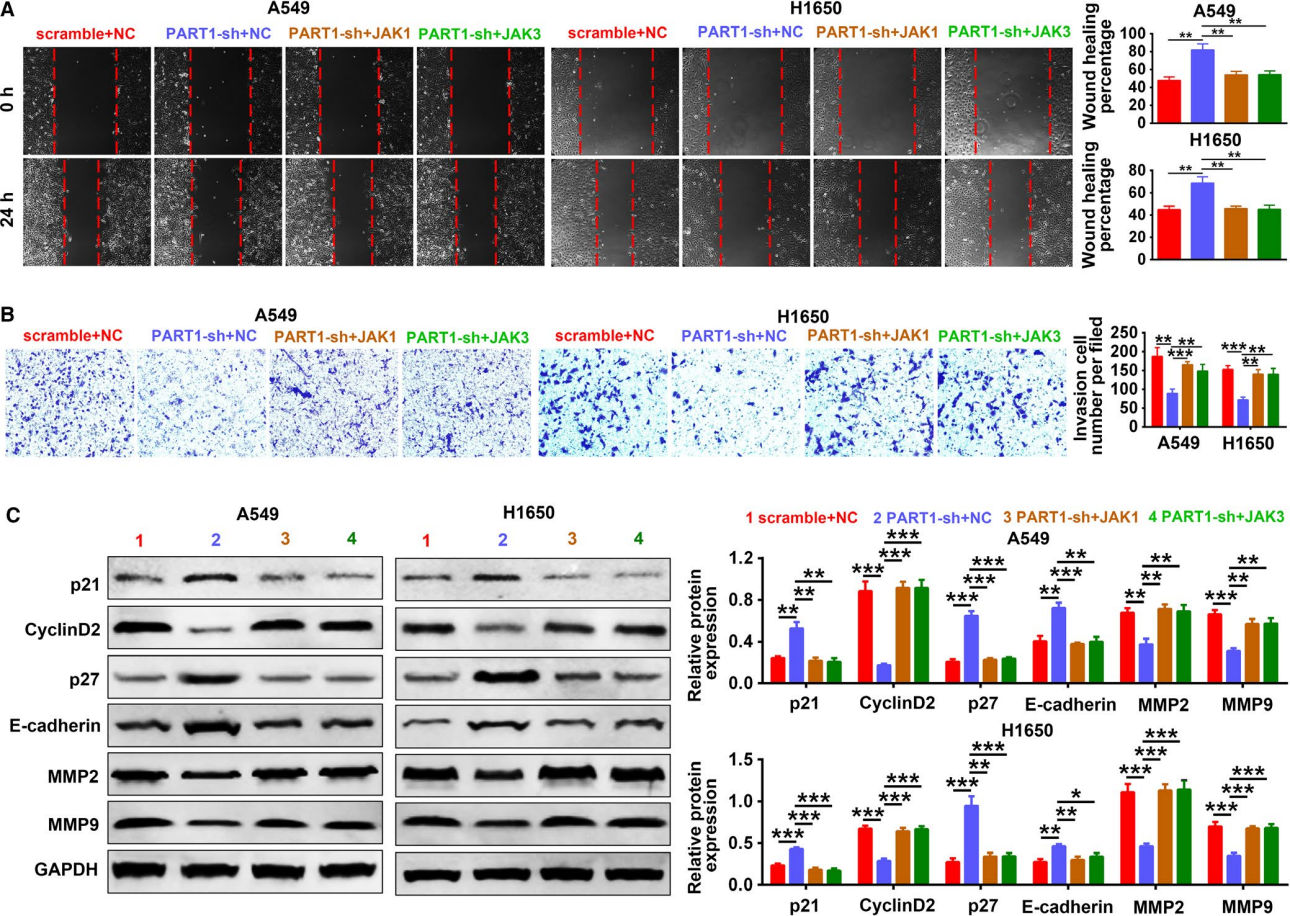
PART1 enhanced cell migration and invasion of NSCLC cells via JAK‐STAT signaling pathway. A, Detection of PART1‐sh and JAK1 or JAK3 on cell migration by wound‐healing assay. **P < .001. B, Detection of PART1‐sh and JAK1 or JAK3 on cell invasion by transwell assay. **, ***P < .01, *P* < .001. C, Detection of PART1‐sh and JAK1 or JAK3 on protein expression of p21, Cyclin D2, p27, E‐cadherin, MMP2, and MMP9 by western blot. *, **, ***P < .05, *P* < .01, *P* < .001. JAK, Janus kinase; NSCLC, non‐small cell lung cancer; PART1, prostate androgen‐regulated transcript 1; STAT, signal transducer and activator of transcription protein

We cotransfected PART1‐sh with pcDNA‐JAK1 or JAK3 into A549 and H1650 cells to determine whether the regulation of PART1 on NSCLC progression was through JAK‐STAT signaling pathway. First, the expression of JAK1, JAK3, p‐JAK1, p‐JAK3, p‐STAT3, TIMP‐1, and Pim‐1 was downregulated by PART1‐sh, while addition of pcDNA‐JAK1 or JAK3 restored the proteins expression (Figure 9A). Second, in line with results of MTT assay (Figure 9B), EdU staining (Figure 9C) and flow cytometry (Figure 9D) showed that the inhibiting ability of PART1‐sh on cell proliferation and cell cycle was restored to the control via addition of pcDNA‐JAK1 or JAK3. Lastly, the migration (Figure 10A) and invasion (Figure 10B) ability inhibited by PART1‐sh were also induced via addition of pcDNA‐JAK1 or JAK3. Moreover, the increased expression of p21, p27, and E‐cadherin was decreased, while the decrease of Cyclin D2, MMP2, and MMP9 was increased by the addition of pcDNA‐JAK1 or JAK3 (Figure 10C). All these data indicated that PART1 enhanced proliferation, migration, and invasion of NSCLC cells via JAK‐STAT signaling pathway.

### 3.8 PART1 knocking down suppressed xenograft tumor growth via inactivating of JAK‐STAT signaling pathway

**Figure 11:**
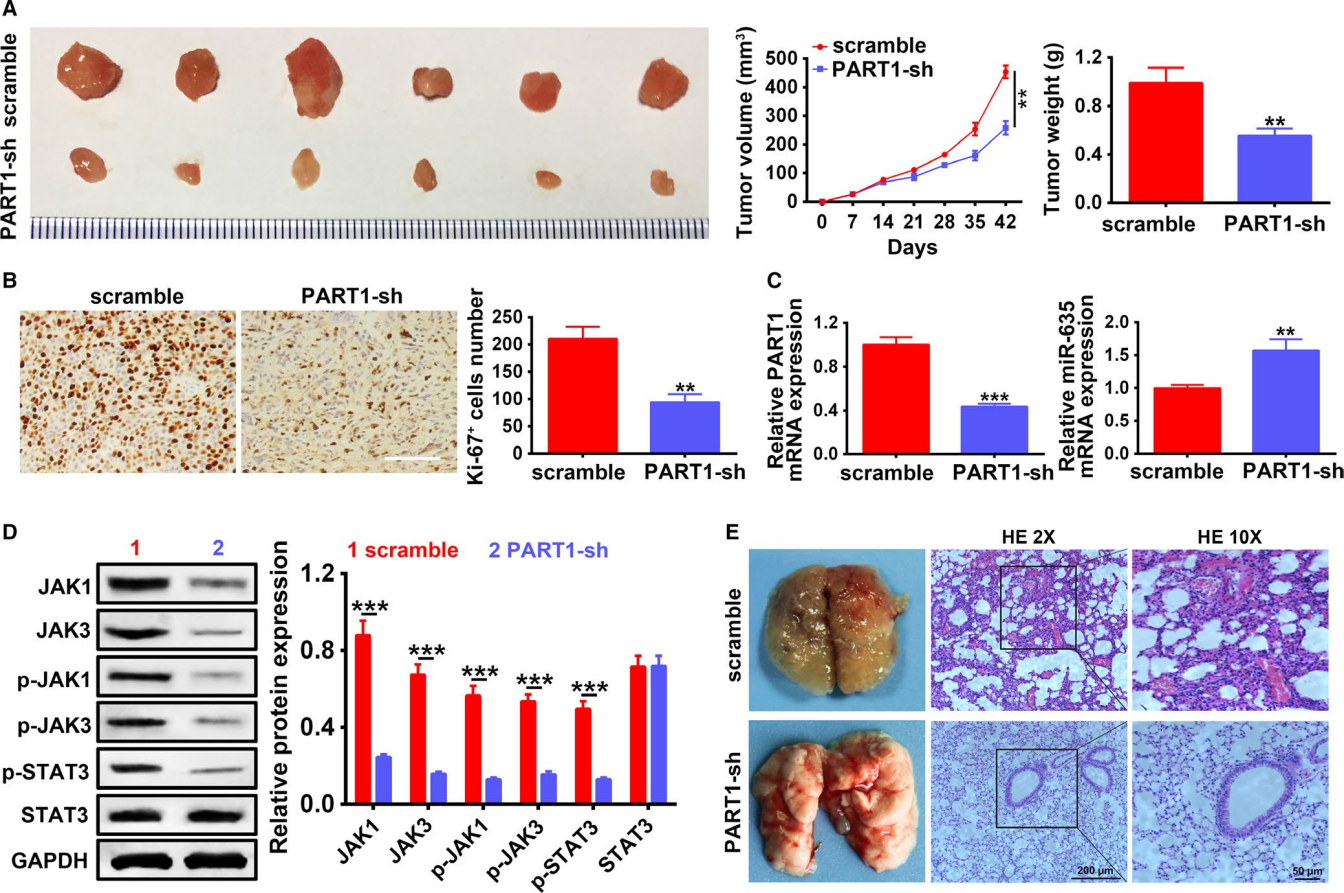
PART1 knocking down suppressed xenograft tumor growth via inactivating of JAK‐STAT signaling pathway. A, Detection of PART1‐sh on tumor growth, volumes, and weights via intratumoral injection of A549 cells with lentiviral vector with PART1 knocking down into xenograft tumor mice model. **P < .01. B, Detection of PART1‐sh on Ki‐67 protein expression and Ki‐67^+^ cells number by immunohistochemistry assay. **P < .01. C, Detection of PART1‐sh on PART1 and miR‐635 expression by qRT‐PCR. **, ***P < .01, *P* < .001. D, Detection of PART1‐sh on JAK1, JAK3, p‐JAK1, p‐JAK3, STAT3, and p‐STAT3 protein by western blot. *** *P* < .001. E, H&E staining of lung nodules formed after intratumoral injection of A549 cells with lentiviral vector with PART1 knocking down into xenograft tumor mice model. Scale bar: 200 μm. H&E, hematoxylin and eosin; JAK, Janus kinase; PART1, prostate androgen‐regulated transcript 1; PCR, polymerase chain reaction; STAT, signal transducer and activator of transcription protein

To further investigate the clinical application of PART1 in NSCLC, we inoculated A549 cells transfected with PART1‐sh into nude mice and detect the effect of PART1 knocking down on xenograft tumor growth. The intratumoral injection of lentiviral vector with PART1 knocking down inhibited tumor growth, wherein the tumor volume and weight were dramatically decreased compared to that of scramble (*P* < .01) (Figure 11A). Ki‐67 protein, cellular marker for cell proliferation, was downregulated in the xenograft tumor tissues injected with PART1‐sh by means of immunohistochemistry (Figure 11B), the number of Ki‐67^+^ cells in the tumor tissues injected with PART1‐sh was also less than that of scramble (Figure 11B). The expression of PART1 was significantly downregulated in the xenograft tumor tissues injected with PART1‐sh (*P* < .001), while miR‐635 was upregulated (Figure 11C). Moreover, we found that the injection of PART1‐sh significantly inhibited JAK1, JAK3, p‐JAK1, p‐JAK3, p‐STAT3 expression (Figure 11D). Full specimen staining with H&E revealed that the xenograft tumor tissues injected with PART1‐sh contained more pulmonary nodules than that of scramble, and reduced the number of lung metastases (Figure 11E). These results suggest that PART1 knocking down suppressed xenograft tumor growth through the mediation of miR‐635 expression and inactivation of JAK‐STAT signaling pathway.

## 4 DISCUSSION

NSCLC is a widespread metastases and poor prognosis tumor.^25^ Recent studies have shown that various types of lncRNAs, such as TBILA,^26^ NNT‐AS1,^27^, ^28^ BLACAT1,^29^ exhibited biological functions and were involved in the progression of NSCLC. Moreover, although PART1 was upregulated in NSCLC specimens with poor prognosis,^14^ the detailed functional role of PART1 in the pathogenesis of NSCLC remains under‐investigated. Our study provided a proof of concept for PART1 as miR‐635 sponging and as molecular regulator of JAK‐STAT signaling pathway, which is the key cellular function relevant to NSCLC.

Genome‐wide gene expression analysis of NSCLC patients revealed that PART1 was induced in squamous cell lung cancer tissues.^30^ In line with the previous study, we first showed that PART1 was upregulated in both human NSCLC tissues and four different NSCLC cell lines, A549, H1650, SK‐MES‐1, and H1975. In addition, we demonstrated the upregulation of PART1 and downregulation of miR‐635 in NSCLC tissues, suggesting negative correlation between them. Genetic changes such as EGFR mutation and epigenetic differentiation as well as the experimental changes like smokers or nonsmokers represent the main pathogenesis of NSCLC,^31^, ^32^, ^33^ which is considered as potential therapeutic schedule for diagnosis, prognosis, clinical follow‐up of NSCLC.^34^ However, both of the expression of PART1 and miR‐635 were not significantly related to the smoking status, tumor differentiation, or EGFR mutations of NSCLC patients in the present study, in line with the previous research.^14^ Therefore, the regulation of PART1 on NSCLC may appear as a brand new mechanism.

The dysregulated expression profiles of PART1 and miR‐635 suggested that PART1 and miR‐635 may participate in the regulation of NSCLC tumorigenesis. RNA pull down confirmed the binding ability between PART1 and miR‐635. Except for miR‐129 in ESCC,^13^ miR‐133a, miR‐135b, miR‐196b, and miR‐193b in oral squamous cell carcinoma,^35^ to the best of our knowledge, we first found that miR‐635 was a new target miRNA for PART1. Moreover, in the present study, we demonstrated that PART1 promoted cell proliferation, migration, and invasion of NSCLC, while knockdown of PART1 performed the opposite effects on NSCLC progression.Other lncRNAs, such as MALAT1,^36^ HOTAIR,^37^ and UCA1,^38^ that were highly expressed in NSCLC, also promoted the progression and the inhibition of these lncRNAs suppressed the progression. Tian et al^39^ found that miR‐635 functioned as tumor suppressor to inhibit tumorigenesis of osteosarcoma. Moreover, miR‐635 was shown to inhibit the tumorigenesis of NSCLC by targeting YY1.^19^ However, in the present study, JAK1 and JAK3 were characterized as the potential targets of miR‐635 by means of dual‐luciferase reporter assay. In NSCLC cell lines, we further found that miR‐635 regulated the protein expression of JAK1 and JAK3. These data revealed that PART1 participated in NSCLC progression via direct targeting miR‐635 and regulating JAK1/JAK3 expression.

The binding of various ligands, such as cytokines, to the cell surface receptors, leads to the dimerization of receptors and thus bringing receptor‐associated JAKs into close proximity.^40^ The JAKs are then activated via phosphorylating each other on tyrosine residues.^40^ The activated JAKs phosphorylate tyrosine residues of STATs and lead to the activation of STATs, thus participating in the regulation of tumor progression.^41^, ^42^, ^43^ JAK1 was shown to activate STAT3 and thus promoting cancer growth in NSCLC,^44^ inhibitor of JAK3‐STAT3 signaling pathway demonstrated potential antitumor activities for NSCLC.^45^ Through the inactivation of JAK‐STAT signaling pathway, knockdown of leptin inhibited cell proliferation and induced apoptosis in NSCLC cells.^46^ Curcumin suppressed NSCLC migration, invasion, and cell proliferation through inactivating JAK‐STAT signaling pathway.^47^Therefore, the effects of PART1/miR‐635 on the expressions of JAK‐STAT signaling pathway related genes (JAK1, p‐JAK1, JAK3, p‐JAK3, STAT3, p‐STAT3, TIMP‐1, and Pim‐1) were measured in NSCLC cell lines. Consistent with those of Yuan et al,^48^ miR‐635 could inhibit the expression of those genes and inactivated JAK‐STAT signaling pathway. Besides, the suppressed role of miR‐635 on JAK‐STAT signaling pathway was abolished via PART1 overexpression. Functional assays further showed that PART1 knockdown‐mediated anti‐proliferation, anti‐migration, and anti‐invasion effects were suppressed via JAK1 or JAK3 overexpression in NSCLC, in line with that of Wang et al^49^ Further xenograft tumor growth model of NSCLC also confirmed the antitumor genesis role of PART1 knocking down via inactivation of JAK‐STAT signaling pathway. Although a large number of lncRNAs are considered as diagnostic and prognostic markers as well as therapeutic targets for NSCLC,^50^, ^51^, ^52^ due to complicated pathogenesis of NSCLC, more molecular mechanisms involved in NSCLC need to be further explored. Moreover, the further study of PART1 on regulation of AS is still indispensable to be investigated via more elaborate animal models.

In summary, the present study demonstrated that lncRNA PART1 knocking down inhibited proliferation, migration, and invasion of NSCLC cells via sponging miR‐635 and inactivating JAK‐STAT signaling pathway. This finding illuminated the relation between PART1/miR‐635/JAK‐STAT regulatory axis NSCLC progression, suggesting potential application of PART1 in treatment for the disease.

## CONFLICT OF INTEREST

The authors declare that they have no competing interests.

## AUTHOR CONTRIBUTIONS

Dengyan Zhu and Yang Yu designed the study. Wei Wang, Kai Wu, and Donglei Liu collected the data, Yang Yang and Chunyang Zhang analyzed the data, Yu Qi analyzed the results and drafted the manuscript, Song Zhao manuscript preparation, final approval.

## Data Availability

The datasets used and/or analyzed during the current study are available from the corresponding author on reasonable request.
